# How Important Is Behavioral Change during the Early Stages of the COVID-19 Pandemic? A Mathematical Modeling Study

**DOI:** 10.3390/ijerph18189855

**Published:** 2021-09-18

**Authors:** Jongmin Lee, Seok-Min Lee, Eunok Jung

**Affiliations:** 1 Mathematics Department, Konkuk University, Seoul 05029, Korea; ljm1729@konkuk.ac.kr; 2 Department of Liberal Arts, Hongik University College of Engineering, Seoul 04066, Korea; thesmlee@gmail.com

**Keywords:** COVID-19, mathematical modeling, nonpharmaceutical intervention, behavioral change, social distancing

## Abstract

How important is the speed and intensity of behavioral change due to government policies, such as enhanced social distancing or lockdown, when an emerging infectious disease occurs? In this study, we introduce a deterministic SEIR model considering the behavior-changed susceptible group to investigate the effect of the speed and intensity of behavioral change on the transmission dynamics of COVID-19. We used epidemiological data from South Korea and Italy for the simulation study, because South Korea and Italy were the first countries to report an outbreak of COVID-19 after China and the prevention and response policy of each government were similar during the first outbreak of COVID-19. Simulation results showed that it took approximately twenty fewer days in Korea than in Italy until 90% of susceptible individuals changed their behavior during the first outbreak. It was observed that the behavior-changed susceptible individuals reduced the COVID-19 transmission rate by up to 93% in Korea and 77% in Italy. Furthermore, if the intensity and speed of behavioral change in Italy were the same as in Korea, the expected number of cumulative confirmed cases would have been reduced by approximately 95%, from 210,700 to 10,700, until the end of the lockdown period. We assumed that behavioral change is influenced by the number of confirmed cases and does not take into account social and cultural differences, as well as the state of the healthcare system, between the two countries. Our mathematical modeling showed how important the high intensity and fast speed of behavioral change to reduce the number of confirmed cases in the early period of an epidemic are.

## 1. Introduction

Since the first report of coronavirus disease 2019 (COVID-19) in Wuhan, China, the worldwide pandemic has been ongoing, and the confirmed cases have been increasing. Despite efforts to end the COVID-19 pandemic globally, the cumulative number of confirmed cases and deaths worldwide as of 10 September 2021 is approximately 220 million and 4.6 million, respectively [1]. Korea and Italy were in the first group of countries reporting the rapid spread of COVID-19, following China. The two countries have similarities in their population size, COVID-19 outbreak period, and phase changes in government intervention policies to strengthen regulations [2]. The populations of Korea and Italy in 2019 were 51,709,098 and 60,302,093, respectively [3]. The first case of local infection was reported on 16 February and 21 February 2020, and the first outbreak of COVID-19 was almost over around 19 April and 3 May 2020, in Korea and Italy, respectively. The mitigation of the first outbreak in the two countries was due to the escalating social distancing policies that rose to the highest level, Social Distancing Level 3 in Korea and lockdown in Italy. The number of cumulative cases by April 19 reached 10,633 in Korea and 178,972 in Italy, which is more than 16-times the cumulative cases in Korea [4,5]. During the first outbreak of COVID-19, the levels of social distancing policies in the two countries were similar. However, the scale of cases was quite different.

Nonpharmaceutical interventions (NPIs) for changing individuals’ behavior in pandemic situations are the primary strategies in the absence of vaccines and medicines [6]. The Homeland Security Council within the Executive Office of the President of the United States has emphasized the impact of individual behavior on the pandemic situation [7,8]. The World Health Organization (WHO) has advised wearing masks and avoiding mass public gatherings to protect one’s self [9]. These efforts to prevent spreading the disease are aimed at ensuring that the number of patients does not surpass the medical capacity, that is these measures are aimed at flattening the curve [10]. The most effective NPIs adopted by most countries’ governments have been social distancing policies, including lockdowns, especially during the early stages [11]. Governmental social distancing policies have appeared to be effective interventions by changing the behavior of individuals. These behavioral changes are supposed to include wearing face masks, washing hands often with soap, cleaning and sanitizing surfaces that are touched frequently, keeping a distance of at least two meters from people, covering coughs and sneezes, not having in-person meetings, staying home if possible, and avoiding crowded places [12].

Mathematical models considering behavioral change have been discussed in several studies. Perra proposed a model that considers behavioral change relative to the incremental confirmed cases [13]. Brauer estimated the reduced contact due to behavioral change using epidemic data [14]. Kim and Jung developed a modified behavioral change model of Perra to explain the spread of COVID-19 in Korea [15]. They also predicted a secondary outbreak by adding an opposite behavioral change term as the number of recovered cases increased [16]. In Mexico, researchers used a SEIR model to study behavioral change and to predict the date of peak incidence occurrence when considering a high level of intervention policies [17]. Hamada estimated the reproductive number using the mobility pattern that represents the behavioral change of a citizen [18]. Most of the modeling studies have highlighted the importance of the intensity of behavioral change in public to mitigate the outbreak of emerging infectious diseases, especially COVID-19. In this study, we used mathematical modeling to investigate the effect of the speed of behavioral change, as well as the intensity of behavioral change on the outbreak size.

The structure of this paper is as follows. In Section 2, the epidemic data and intervention policies in Korea and Italy are introduced. The behavioral change model of COVID-19 is then presented, and the parameters implying the intensity and speed of behavioral change are introduced in this section. In Section 3, simulations of various scenarios, including the condition that the behavioral change in Italy was at a similar level to that of Korea, are investigated. Discussions are presented in the final section.

## 2. Materials and Methods

### 2.1. Epidemic Data

**Figure 1:**
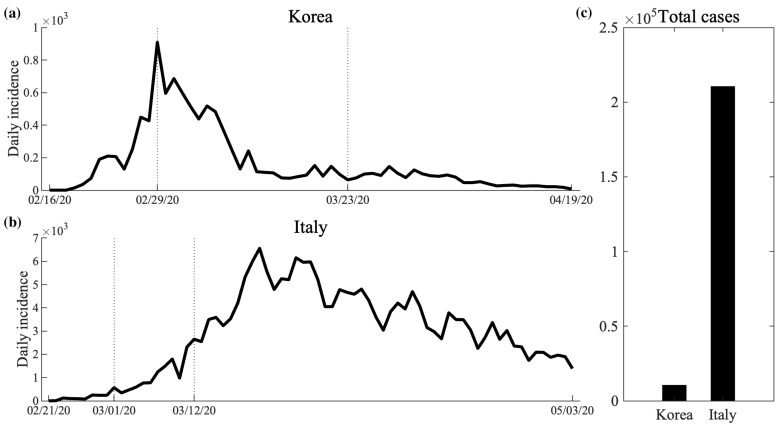
Confirmed cases during the first outbreak of COVID-19 in Korea and Italy: (**a**) daily confirmed cases in Korea (16 February 2020∼19 April 2020); (**b**) daily confirmed cases in Italy (21 February 2020∼5 March 2020); (**c**) cumulative confirmed cases in Korea and Italy at the end of the simulation period.

Daily COVID-19 data were aggregated from the Korea Disease Control and Prevention Agency (KDCA) and the Italian National Institute of Health (ISS) [4,5]. Since we considered community transmission during the first outbreak of COVID-19, the confirmed cases before the initial community spread were ignored: 28 cases before 16 February in Korea and 3 cases before 21 February in Italy. The simulation periods were set to be the time from the first community spread to the end of the enhanced social distancing strategy or lockdown: from 16 February to 19 April 2020 in Korea and from 21 February to 3 May 2020 in Italy. During this first outbreak period, the numbers of cumulative cases in Korea and Italy were 10,634 and 210,714, respectively, with peaks on 28 February 2020 (909 cases) and 3 March 2020 (6557 cases). Figure 1 displays the daily confirmed cases in Korea (a) and Italy (b) during the simulation period. The cumulative confirmed cases at the end of the period are shown in (c). In (a) and (b), the vertical dotted lines represent the changes in the political intervention strategy described in Section 2.2.

### 2.2. Social Distancing Strategies in Korea and Italy

We classified the government intervention policies taking into account the level of social distancing based on [4,19]. In Korea, the national alert level was set to red, the highest level, from orange for COVID-19 on 23 February 2020, after the local community spread began on 16 February. Social Distancing Level 2 began on 29 February, when the first outbreak peaked. From 23 March to 19 April, enhanced social distancing was implemented. The details of each policy in Korea were explained in [20].

On 21 February, clusters of cases by community spread occurred in Lombardy and Veneto. Regional restrictions were added on 1 March, as the number of confirmed cases increased rapidly in the northern provinces [21]. As COVID-19 spread across the country, the intervention measures in 14 northern regions were expanded nationwide from 10 March, and restrictions were strengthened and enforced from 12 March [22]. The period from 12 March to 3 May was the period of lockdown, the strongest intervention policy.

**Figure 2:**
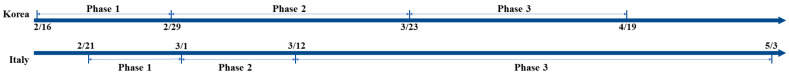
Timeline of COVID-19 intervention policies in Korea (**top**) and Italy (**bottom**).

Korea and Italy showed similar intervention policies for COVID-19 in the early stages of the first outbreak. Both countries immediately declared an epidemiological emergency as regional transmission expanded. As the number of confirmed cases increased rapidly across the country, strong social distancing interventions were implemented. Based on these similarities, we used the following political phases in this study: Phase 1 started from the day that community spread began. In Phase 1, the government announced a red national alert and political interventions, such as border screening and 14 d quarantine. In Phase 2, both countries enforced social distancing or regional restrictions (Social Distancing Level 2). Phase 3 was the period of the strongest intervention policies (Social Distancing Level 3 or lockdown). Figure 2 shows the timeline of each country’s political intervention phases for COVID-19.

### 2.3. Mathematical Model Considering Behavioral Change

The mathematical model considering the behavioral change introduced in this study is a modified version of the previous works [13,15]. It is a modified SEIQR model, based on the widely used deterministic SEIR model added by the isolated group *Q*. The total population is divided into six groups: normal susceptible group (*S*) and behavior-changed susceptible group ($S_{F}$), which are two groups of susceptible individuals to COVID-19, the latent group (*E*) that is exposed and infected, but can not yet infect other people, the infectious group (*I*) that can infect others, the confirmed and subsequently isolated group (*Q*), and the recovered group (*R*) that is released from quarantine. It was assumed that an infectious individual would be isolated immediately after infection was confirmed and would not come into contact with susceptible individuals. Due to this, the population having a chance to come into contact with others, *N*, is the sum of *S*, $S_{F}$, *E*, *I*, and *R*.

Parameter β denotes the transmission rate at which a normal susceptible individual is infected by contact with an infectious individual and becomes an exposed and latent individual. Those in group $S_{F}$ are behavior-changed susceptible individuals with a fear of disease who have a relatively low transmission rate (δβ) of COVID-19 due to behavioral change, where δ is the reduction ratio of transmission in the behavior-changed group. The parameter $\beta_{F}$ is the number of people who changed their behavior per the number of daily incidence. The parameters κ and α are, respectively, the mean progression rate and isolation rate. Therefore, 1/κ and 1/α represent the mean latent period and infectious period from being able to infect other people to isolation, respectively. Parameter γ is the mean recovery rate, and *f* is the mean case fatality ratio (CFR). The mean recovery period (1/γ) was estimated as 20.7 d based on the data reported by the KDCA [23]. Parameters β, $\beta_{F}$, and δ were estimated from the data, and we also found a 95% confidence interval (CI). Table 1 shows all parameters of the behavior-changed model of COVID-19.

**Figure 3:**
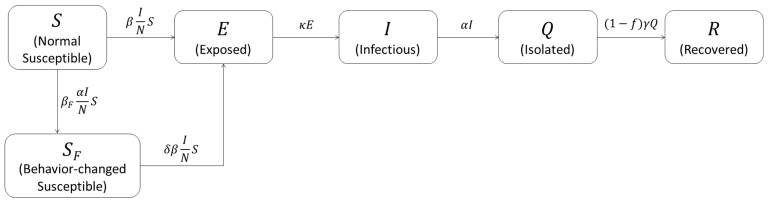
Flow diagram of the behavior-changed model.

The main feature of the behavior-changed model in this study is that the susceptible people were divided into two groups: *S* and $S_{F}$. The individuals in group $S_{F}$ feel fear proportional to the daily confirmed cases (αI). Note that $\beta_{F}$ represents the speed of behavioral change due to the increasing number of daily confirmed cases and the enhanced government policy. Then, the behavioral change term, a flow from *S* to $S_{F}$, was set to $\beta_{F}\left(\alpha I\right)\frac{S}{N}$, as a function of the number of confirmed cases αI. Another parameter indicating behavioral change in people is δ, which represents the level of intensity of behavioral change. Figure 3 shows the flow diagram of the COVID-19 model with behavioral change described by the ordinary differential equations (ODEs) as follows.

(1) $$\begin{array}{ccc} \frac{dS}{dt} & = & -\beta \frac{I}{N}S-\beta_{F}\alpha I\frac{S}{N}, \end{array}$$

(2) $$\begin{array}{ccc} \frac{dS_{F}}{dt} & = & \beta_{F}\alpha I\frac{S}{N}-\delta \beta \frac{I}{N}S_{F}, \end{array}$$

(3) $$\begin{array}{ccc} \frac{dE}{dt} & = & \beta \frac{I}{N}S+\delta \beta \frac{I}{N}S_{F}-\kappa E, \end{array}$$

(4) $$\begin{array}{ccc} \frac{dI}{dt} & = & \kappa E-\alpha I, \end{array}$$

(5) $$\begin{array}{ccc} \frac{dQ}{dt} & = & \alpha I-\gamma Q, \end{array}$$

(6) $$\begin{array}{ccc} \frac{dR}{dt} & = & (1-f)\gamma Q. \end{array}$$

The initial values were determined as follows: since confirmed cases were isolated after 1/α d of being infectious, the number of confirmed cases within 1/α d from the start of the simulation was assumed to be $I_{0}$. In addition, since confirmed cases were isolated on average 1/α+1/κ d after exposure to the virus, the number of confirmed cases during 1/κ d after 1/α d was assumed to be $E_{0}$. It was also assumed that there was no population with behavioral change at the beginning of the simulation, and there were no cured people and no deaths. As a result, ($I_{0}$, $E_{0}$) in Korea and Italy are (317, 417) and (884, 806), respectively.

### 2.4. Sensitivity Analysis: PRCC-LHS

Sensitivity analysis is a tool used to identify which input parameters are the most influential to the model output, which in our case is the cumulative confirmed cases. In this study, we used the partial rank correlation coefficient (PRCC) method combined with the Latin hypercube sampling (LHS) technique for the sampling of the parameters. The PRCC-LHS method is one of the most reliable global sensitivity analysis techniques [28]. We calculated the PRCC values for the parameters β, α, $\beta_{F}$, δ, κ, and γ. We sampled 10,000 parameter combinations from uniform distributions [29,30,31,32,33,34]. The time points were chosen every 7 d from the second week of the simulations. Positive and negative PRCC values mean that the number of cumulative confirmed cases increases and decreases, respectively, as the value of the input parameter increases. A higher magnitude of a PRCC value indicates a greater influence of the input parameter on the model output.

## 3. Results

### 3.1. Data Fitting Result

**Figure 4:**
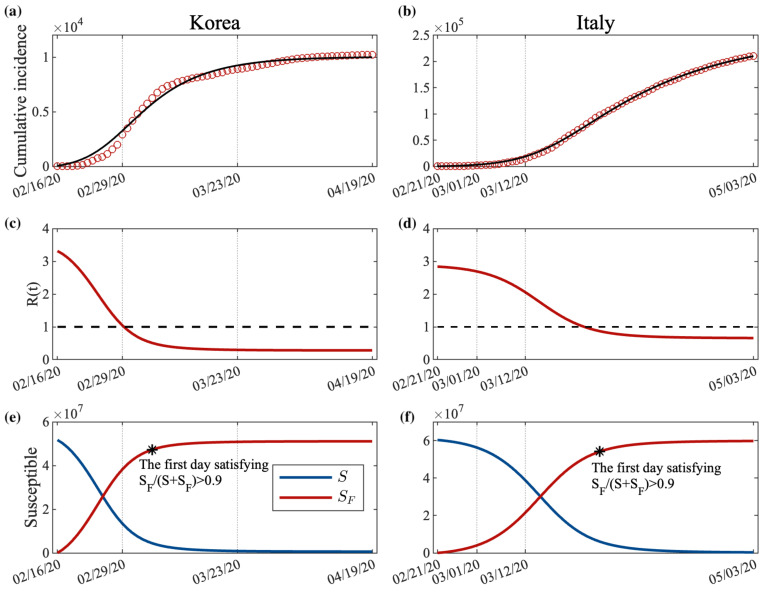
Data fitting result for Korea and Italy: (**a**,**b**) cumulative confirmed data and behavior-changed model result; (**c**,**d**) reproductive number derived by the behavior-changed model; (**e**,**f**) population of normal susceptible and behavior-changed susceptible. The star is the first day satisfying $S_{F}/\left(S+S_{F}\right)>0.9$.

Figure 4a,b displays the best-fit model curves and the reported data for the cumulative confirmed cases in Korea and Italy, respectively. The parameters β, $\beta_{F}$, and δ were estimated using a least-squares fitting method, lsqcurvefit in the MATLAB functions. The estimated and data-fit parameter values in Korea and Italy are given in Table 1. The transmission rate β in Korea was estimated to be higher than that in Italy. However, δ in Korea was lower than in Italy, resulting in the transmission rate in $S_{F}$, δβ, in Korea being lower than that in Italy. The transmission rate of the behavioral change group in Italy was approximately 0.09, which was estimated to be approximately 2.25-times higher than the 0.04 in Korea. The number of people who changed their behavior from *S* to $S_{F}$ per the number of daily incidence, $\beta_{F}$, in Korea was estimated to be approximately 15-times larger than that in Italy.

Figure 4c,d shows the reproductive numbers, R(t), which can be calculated by the estimated parameters. Details are attached to the Appendix A. At the beginning of the outbreak, the values of R(t) were quite high in both countries; R(t) was approximately 3.31 in Korea and 2.84 in Italy. In Korea, R(t) decreased rapidly, reached 1 on 29 February, and then stayed at approximately 0.28 until the end of the simulation period. An interesting observation is that people changed their behavior earlier than the government’s enhanced social distancing policy that began on 23 March 2020. In Italy, R(t) also declined smoothly and reached 1 on 26 March, then remained at approximately 0.66 until the end of lockdown.

Figure 4e,f displays the normal susceptible (blue) and behavior-changed susceptible (red) groups as a function of time in Korea and Italy during the early outbreak of COVID-19, respectively. The star indicates the day when more than 90% of susceptible individuals changed their behaviors due to an increase in new confirmed cases or government policies, such as social distancing and lockdown. We refer to the time for 90% of the susceptible population to change their behavior as behavioral change timing (BCT). BCT in Korea and Italy was observed at 20 d (Phase 2) and 39 d (Phase 3), respectively, after the start of the simulation. The peak time and cumulative confirmed numbers in Korea were 16 d earlier and 95% less than those in Italy.

### 3.2. Sensitivity Analysis

**Figure 5:**
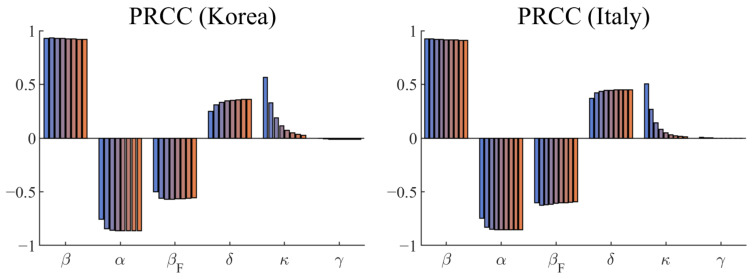
PRCC values depicting the sensitivities of the confirmed cumulative cases, with respect to the model parameters β, α, $\beta_{F}$, δ, κ, and γ.

Figure 5 shows the PRCC values depicting the sensitivities of the model output, ∫αIdt, with respect to the parameters β, α, $\beta_{F}$, δ, κ, and γ. Observe that β, δ, and κ were positively correlated with the model output, while α and $\beta_{F}$ were negatively correlated. Note that the PRCC values for γ were close to zero because γ did not affect the model output. The sensitivities of the parameters in both countries were similar at all times. The parameter β was the most sensitive, followed by α, $\beta_{F}$, δ, and finally, by κ. At the end of the simulation, the PRCC values of β, α, $\beta_{F}$, δ, and κ in Korea and Italy were (0.92, 0.91), (−0.86, −0.85), (−0.56, −0.60), (0.36, 0.45), and (0.03, 0.01), respectively. In Italy, $\beta_{F}$ and δ were slightly more sensitive than in Korea. Numerical simulations of the sensitivity analysis were performed using the PRCC-LHS script presented in [28].

### 3.3. Analysis of Behavioral Change

**Figure 6:**
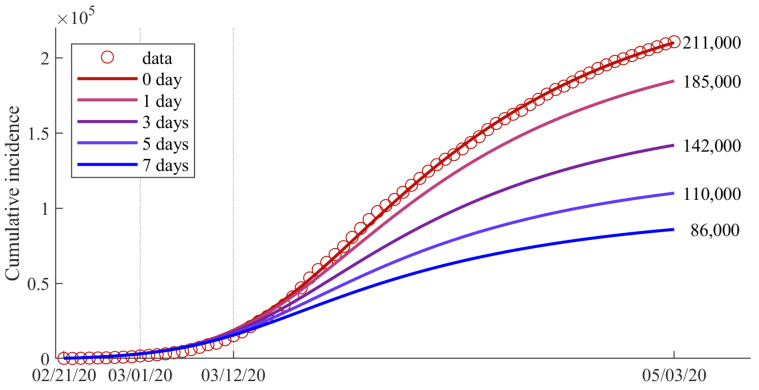
Expected number of cumulative confirmed cases when BCT was advanced by *n d*, for *n* = 0, 1, 3, 5, and 7.

In this subsection, we investigate the effect of the speed and intensity of behavioral change on the epidemic size. To estimate the impact of the faster behavioral change in Italy, the parameter $\beta_{F}$ was varied. We varied $\beta_{F}$ to a value that made BCT *n* d faster. As $\beta_{F}$ increased, BCT also increased. Figure 6 shows the expected number of cumulative confirmed cases as $\beta_{F}$ changed (corresponding *n* = 0, 1, 3, 5, 7 d). The red circle and solid curve (default) represent the data and the best fitted curve in Italy, respectively. If the behavioral change in the susceptible group occurred quickly so that BCT was earlier by 1 d, the number of cumulative confirmed cases would be expected to decrease by 12.3% to approximately 26,000. If BCT was started 7 d earlier, the expected number of cumulative incidences on May 3 would have decreased by 59.2% to approximately 125,000. Note that $\beta_{F}$ was adjusted to 1.15- and 2.65-times when BCT was advanced by 1 d and 7 d, respectively.

**Figure 7:**
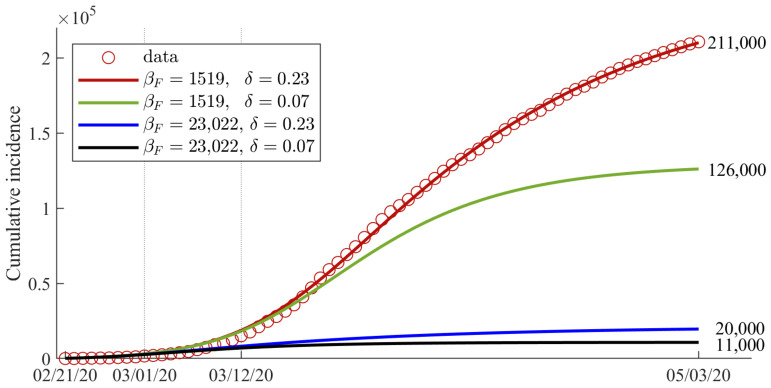
Expected number of cumulative confirmed cases if individuals in Italy had changed their behaviors faster and more intensely.

Figure 7 shows the expected number of cumulative confirmed cases in Italy according to various values of $\beta_{F}$ and δ. The red circle and solid curve (default) represent the data and the best-fit curve in Italy. The green solid curve shows the case in which the behavior-changed group in Italy has the same δ value as that in Korea. The blue solid curve shows the case in which the speed of behavioral change, $\beta_{F}$, in Italy is the same as that in Korea. The black solid curve shows the case in which the values of $\beta_{F}$ and δ in Italy are the same as the values in Korea. If individuals in Italy changed their behaviors faster and more intensely (black curve), the expected number of cumulative confirmed cases might have been reduced by approximately 95%, from 211,000 to 11,000, through the end of the lockdown period. Simulation results showed that the intensity and the speed of behavioral change were important to reduce the number of confirmed cases, especially during the early outbreak period. Note that because our model does not take into account the social, cultural, or medical system, which may differ from country to country, and assumes that people’s behavior changes as a function of the number of confirmed cases, there may be limitations in the interpretation of the results.

## 4. Discussion

In this study, using mathematical modeling, we investigated the impact of behavioral change due to enhanced social distancing and fear of disease on the transmission dynamics of the COVID-19 pandemic during the early stages of the outbreak in Korea and Italy. At the time of the first outbreak in Korea, large-scale mass infections from certain religious groups or medical care hospitals were concentrated in the Daegu and Gyeongbuk regions. Meanwhile, the first outbreak in Italy was concentrated in the northern regions near Lombardy, mainly in nursing homes. We considered community transmission of COVID-19 in the simulation period from 16 February to 19 April 2020 for Korea and from 21 February to 3 May 2020 for Italy.

We introduced the susceptible group with behavioral change ($S_{F}$) and the intensity and speed of behavioral change (δ and $\beta_{F}$) in our model. With respect to the impact of behavioral change, there was a significant relationship between the parameters δ and $\beta_{F}$ and the size of the outbreak. The reduction ratio of transmission in the behavior-changed group was estimated as δ=0.07 for Korea and δ=0.23 for Italy if $\beta_{F}$ were assumed as the same in both countries. It was observed that the intensity of behavioral change in Italy was three-times weaker than that in Korea. The transmission rate in the behavior-changed susceptible group, δβ, was 0.04 for Korea and 0.09 for Italy. If δ in Italy were reduced to 0.07, the number of confirmed cases would have reduced by approximately 85,000 (40.3% reduction). Many papers have discussed the importance of the intensity of social distancing [35,36,37].

Our simulation results showed that the speed of behavior change is also important to reduce the number of confirmed cases and prevent the spread of emerging diseases such as COVID-19, especially during the early outbreak period. As can be seen from the simulation results (Figure 4e,f), BCT took approximately 20 d in Korea and 39 d in Italy. Furthermore, if the behavioral change in Italy had occurred quickly, the number of cumulative confirmed cases would have decreased by approximately 26,000 (12.3% reduction) if BCT were 1 d earlier and more than 125,000 (59.2% reduction) if BCT were a week earlier. Furthermore, if the speed of behavioral change, $\beta_{F}$, in Italy were similar to that in Korea, then the number of cumulative incidences would be reduced from 211,000 to 20,000 (about 90.5% reduction) (see the blue curve in Figure 7). If both the intensity and speed of behavioral change were the same as in Korea, the total number of confirmed cases until 3 May would have been approximately 11,000 (94.8% reduction). As in our study, Alagoz, et al. claimed that an early social distancing policy could effectively reduce the number of cumulative confirmed cases [38].

We also estimated the reproductive numbers in Korea and Italy with continuous decreasing functions due to the shift from the normal susceptible group to the behavior-changed susceptible group. The slope of the reproductive number in Korea was much steeper than that in Italy. The reproductive number reached the critical value after 13 d from the start of simulation in Korea, that is 23 d earlier than Italy. In Korea, a policy to enhance social distancing was introduced on 23 March 2020; however, interestingly, people changed their behaviors faster than the government’s policy (see Figure 4c–f).

There were some limitations in our mathematical modeling study. First, asymptomatic infectious people were not considered due to the lack of data on the proportion of asymptomatic infectious people and their infectivity during the first outbreak of COVID-19 in Korea. This may have led to overestimating the transmission rate or shortening the duration of infection. Second, the model needs to be modified when considering the second or third wave of COVID-19. In our model, there is no back flow from the behavior-changed susceptible group to the eased susceptible group because we only considered the first outbreak period of COVID-19 in this study. Lastly, although there may be various reasons for behavioral change, such as culture, economy, social activity, and health care systems in different countries, it was assumed in our model that behavioral change is caused by fear or awareness of the increase in the number of confirmed cases. Therefore, there are limitations to consider when interpreting the results. The purpose of this study was not to compare the two countries, but to emphasize the importance of the intensity and speed of behavioral change through mathematical modeling. Despite these limitations, the behavior-changed model of COVID-19 can provide important features in determining proactive and effective intervention policies for COVID-19, especially during the early period of the outbreak.

## 5. Conclusions

When an outbreak of an emerging infectious disease spreads, it is important to control the number of patients to protect the health care system through NPIs as it takes approximately 1 y for a treatment or vaccine to be developed. We emphasized that the rapid enforcement of NPIs, such as social distancing, by citizens and governments can effectively reduce the number of new cases. This study showed the importance of rapid and strong behavioral change in reducing the transmission of emerging infectious diseases such as coronavirus disease 2019 by analyzing the speed and intensity of behavioral change through mathematical modeling during the early stages of a pandemic. In this study, behavioral change was assumed to be influenced by the number of confirmed cases. Differences in the cultural and social behavior and the state of the healthcare system among different countries can be included in future studies. The reduction due to rapid and strong behavioral change can be achieved through the quick response of citizens and governments: active contact tracing and changing personal behavior, such as maintaining personal hygiene, wearing masks, washing hands, and refraining from meetings.

## Figures and Tables

**Table 1:** Parameter values in Korea and Italy.

Parameters		Korea		Italy	
**Symbol**	**Description**	**Value (CI)**	**References**	**Value (CI)**	**References**
$\beta_{F}$	Number of people who changed their behavior per the number of daily incidence	23,000 (15,000, 31,000)	data-fit	1500 (1400, 1600)	data-fit
β	Transmission rate in normal susceptible group (per day)	0.55 (0.49, 0.61)	data-fit	0.40 (0.39, 0.40)	data-fit
δ	Reduction ratio of transmission in the behavior-changed group	0.074 (0.023, 0.13)	data-fit	0.23 (0.023, 0.13)	data-fit
1/κ	Latent period (days)	2.1	[24,25]	2.1	[24,25]
1/α	Infectious period (days)	6	[25,26]	7.16	[25,27]
1/γ	Isolation period (days)	20.7	[23]	20.7	[23]
*f*	Case fatality ratio	0.022	[4]	0.14	[5]

## Data Availability

The number of daily confirmed cases in Korea and Italy was aggregated from the Korea Disease Control and Prevention Agency (KDCA) and Italian National Institute of Health (ISS) (Korea: https://www.kdca.go.kr/index.es?sid=a3, Italy: https://github.com/pcm-dpc/COVID-19/tree/master/dati-regioni, accessed on 17 September 2021).

## Abbreviations

The following abbreviations are used in this manuscript:

COVID-19	Coronavirus disease 2019
NPIs	Nonpharmaceutical interventions
WHO	World Health Organization
KDCA	Korea Disease Control and Prevention Agency
ISS	Italian National Institute of Health
CFR	Case fatality ratio
PRCC	Partial rank coefficient correlation
LHS	Latin hypercube sampling
BCT	Behavior change timing

## Appendix A. Reproductive Number R(t)

The reproductive number represents the average number of secondary cases produced by a single primary patient during the period of infection, and is typically computed using the next generation method [39,40]. We set $x={\left(x_{1},x_{2},x_{3},x_{4},x_{5},x_{6}\right)}^{T}={\left(E,I,Q,S,S_{F},R\right)}^{T}$ as the vector of state variables. The compartments $x_{1},x_{2}$, and $x_{3}$ contain infected individuals. For i=1,2,3, we consider $\frac{dx_{i}}{dt}=F_{i}\left(x\right)-V_{i}\left(x\right)$, where $F_{i}\left(x\right)$ is the rate of new infections in the *i*-th compartment and $V_{i}\left(x\right)$ is the rate of the other transitions. Two matrices $F=\left[\frac{\partial F(x_{0})}{\partial x_{j}}\right]$ and $V=\left[\frac{\partial V(x_{0})}{\partial x_{j}}\right]$ for 1≤i,j≤3 are defined, where $x_{0}$ is the state free of infections. The matrix $FV^{-1}$ is the next generation matrix, and its spectral radius, or its maximal real eigenvalue, results in the reproductive number. In this model, the calculation is as follows:

$$\begin{array}{c} F=\left(\begin{array}{ccc} 0 & \frac{\beta (S+\delta S_{F})}{N} & 0\\ 0 & 0 & 0\\ 0 & 0 & 0 \end{array}\right),\;V=\left(\begin{array}{ccc} \kappa & 0 & 0\\ -\kappa & \alpha & 0\\ 0 & -\alpha & \gamma \end{array}\right),\;and\\ FV^{-1}=\left(\begin{array}{ccc} \frac{\beta (S+\delta S_{F})}{\alpha N} & \frac{\beta (S+\delta S_{F})}{\alpha N} & 0\\ 0 & 0 & 0\\ 0 & 0 & 0 \end{array}\right) \end{array}$$

where $N=S+S_{F}+E+I+R$. Finally, we obtain $R\left(t\right)=\frac{\beta (S+\delta S_{F})}{\alpha N}$.
